# Female sterilization paradoxical association with premature menopause in Bihar

**DOI:** 10.1038/s41598-025-04284-x

**Published:** 2025-06-04

**Authors:** Karan Babbar

**Affiliations:** Jindal Global Business School, OP Jindal Global University, Sonipat, India

**Keywords:** Premature menopause, Menopause, Female sterilization, Hysterectomy, Bihar, NFHS-5, Public health, Epidemiology

## Abstract

Premature menopause is a growing public health crisis with serious implications for women’s well-being. While premature menopause prevalence varies across India, Bihar’s rates are exceptionally high (11%), warranting specific investigation. Analyzing data from National Family Health Survey-5, this study examines state-level variations and predictors of premature menopause, focusing on the context of Bihar. Bihar’s premature menopause rates were significantly higher than in other states, even after controlling for hysterectomy, suggesting unique regional drivers. While lower education, more children, and younger age at first and last birth were risk factors across India, this is especially concerning in Bihar. I found a strong association between female sterilization and both naturally occurring and hysterectomy induced premature menopause in Bihar, despite its intended role in family planning. This paradoxical finding, coupled with the protective effect of contraceptive use, raises critical questions about the potential unintended consequences of sterilization practices in Bihar and highlights the need for comprehensive reproductive healthcare services. The study underscores the urgent need for targeted public health interventions, including investments in girls’ education, expanded reproductive healthcare options beyond sterilization, improved sterilization practices through stricter regulation and provider training, and further investigation into the complex factors driving Bihar’s high premature menopause rates.

## Introduction

Imagine experiencing menopausal symptoms such as *hot flashes, mood swings, sleep disturbances*, not in your 50 s or 60 s, but in your 30 s. Premature menopause, the cessation of menstruation before the age of 40^1^, is a growing global health concern with potentially severe implications for women’s long-term well-being. Affecting an estimated 2 to 4% of women worldwide^2^, premature menopause is not simply an early onset of expected biological changes; it is associated with increased risks of cardiovascular disease due to the loss of estrogen’s protective effects on the heart and blood vessels, osteoporosis due to accelerated bone loss, and a range of other health complications, including cognitive decline, mood disorders, and sexual dysfunction^3,4^. These health risks not only impact individual well-being but also pose a significant burden on healthcare systems, particularly in resource-constrained settings. Premature menopause, as a critical juncture in women’s reproductive lives, underscores the intricate relationship between gender inequality and health disparities.

Within India, the prevalence of premature menopause varies significantly across states, with some regions experiencing rates far exceeding national averages^5,6^. Even more concerning is the state of Bihar, where alarmingly high rates of premature menopause persist, even after accounting for the influence of hysterectomies, a finding that distinguishes Bihar from other high-prevalence states^6,7^ and demanding further investigation. Specifically, this study investigates the following research questions: (1) What factors contribute to the high prevalence of premature menopause in Bihar compared to other Indian states? (2) Is there a significant association between female sterilization and premature menopause in Bihar, even after controlling for known risk factors?

Bihar is one of India’s most socioeconomically disadvantaged states^8^. These socioeconomic challenges, coupled with limited access to quality healthcare, demonstrates the complex interplay of social determinants of health and biological factors that contribute to premature menopause. These intersecting vulnerabilities may explain the state’s disproportionately high rates of premature menopause. However, existing research on menopause in India has often overlooked the unique challenges faced by women in specific states like Bihar, particularly regarding the drivers of high premature menopause rates. While recent studies have examined premature menopause at a national level^6,7^, none have focused on the distinct factors contributing to the high prevalence in Bihar. This study builds upon previous national-level analyses by providing an in-depth examination of Bihar^6,7^, aiming to uncover the complex interplay of factors that may be driving the state’s disproportionately high premature menopause rates, particularly the potential role of female sterilization. I consider the potential for both direct and indirect effects of sterilization, including the ethical dimensions of sterilization practices and potential complications leading to hysterectomy.

This study aims to address this gap by examining the factors contributing to Bihar’s uniquely high rates of premature menopause. Using data from the 2019–21 National Family Health Survey-5 (NFHS-5), I analyze a range of sociodemographic, reproductive, and health-related factors associated with premature menopause in Bihar, comparing them to national averages and other high-prevalence states. By identifying the key drivers in this region, this research seeks to inform the development of targeted public health interventions aimed at improving women’s health and well-being in Bihar. These findings contribute to the growing body of literature on menopause in India by highlighting the exceptionally high rates in Bihar and the urgent need for context-specific interventions^6,7,9–12^. I build upon previous work by providing an in-depth analysis of Bihar, uncovering the complex interplay of factors that may be contributing to the state’s disproportionately high premature menopause rates.

## Methods

### Data

This study uses the data from the Indian version of Demographic Health Survey, also known as National Family Health Survey-5 (*NFHS-5*), conducted in India between 2019 and 2021. The NFHS-5 uses a stratified, multi-stage cluster sampling design to ensure national representativeness, it collects comprehensive information on health and demographic indicators, including women’s reproductive health. This study focused on women aged 30 to 39 who were currently not pregnant or non-amenorrhoeic or those who have not menstruated since their last birth^6,10^. I analyzed a subset of 191,087 women aged 30 to 39 from the women’s questionnaire who were neither pregnant nor had menstruated since their last birth—criteria designed to identify potential cases of premature menopause. The NFHS-5 data collection followed ethical guidelines and obtained informed consent from all participants.

### Measures

#### Dependent variable

Premature menopause is defined as the cessation of menstruation for 12 consecutive months or more before the age of 40. Women were classified as experiencing premature menopause if they: (a) self-reported currently being in menopause, (b) had undergone a hysterectomy, or (c) met the criteria for amenorrhea, outlined by Babbar & colleagues^6^. It’s important to note that the NFHS-5 data does not provide information on the specific type of hysterectomy performed (i.e., whether the ovaries were removed—oophorectomy—or not).

#### Independent variable

Guided by previous research^6,7,9,10^, I included a range of sociodemographic, reproductive, and health-related factors as potential predictors of premature menopause. These factors included:

Sociodemographic characteristics: Education level (No education, Primary, Secondary, Higher), Wealth Index (Poor, Middle, Rich), Caste (Scheduled Caste, Scheduled Tribe, Other Backward Classes, General, Don’t Know), Religion (Hindu, Muslim, Others), and area of residence (Urban, Rural).

Reproductive history: Age Group (30–34, 35–39), Number of children ever born (0, 1–2, 3–4, 5+), Age at first and last birth (< 18, 18–21, 22–25, 26–29, 29+) and Contraceptive Use (No, Yes).

Health indicators: Body Mass Index (BMI) (< = 18.5 kg/m^2^, 18.5–24.9 kg/m^2^, > 25 kg/m^2^), Anemia (No, Yes), History of female sterilization (No, Yes), and Health insurance (No, Yes).

### Data analysis & summary statistics

I used binary logistic regression to estimate the odds of experiencing premature menopause in Bihar compared to other states, and to identify factors associated with premature menopause via the two primary pathways to premature menopause: naturally induced and hysterectomy induced. The dependent variable for all models was binary, indicating the presence or absence of premature menopause (hysterectomy/ naturally induced menopause). All models adjusted for sociodemographic factors (*education, wealth, caste, religion, area of residence*), reproductive history (*age group, number of children ever born, age at first and last birth, contraceptive use*), and health indicators (*BMI, anemia, history of female sterilization, health insurance*). District fixed effects were included in all models to account for unobserved variations at the district level. All analyses were conducted using Stata v.18, and standard errors were clustered at the district level.

## Results

Descriptive analysis reveals a concerning prevalence of premature menopause among women in India, particularly in Bihar. As shown in Table 1, approximately 15% of currently married women aged 30–49 across India are already in menopause, with rural areas (*16%*) experiencing slightly higher rates than urban areas (*12%*). Notably, several states report menopause prevalence exceeding the national average, signaling a potential public health crisis.

**Table 1:** Percentage of currently married women aged 30–49 in Menopause by age, NFHS-5 (2019–21).

India/State	Age							
	30–34	35–39	40–41	42–43	44–45	46–47	48–49	Overall
India-Urban	1.23	3.74	8.99	12.98	21.71	30.14	46.14	12.19
India-Rural	2.96	6.43	13.3	18.14	28.13	37.1	50.62	15.96
India-Total	2.36	5.5	11.81	16.33	25.96	34.64	49.1	14.66
State								
Andhra Pradesh	5.47	11.11	24.34	27.21	38.89	49.69	63.31	23.94
Telangana	5.02	12.11	21.02	29.34	40.92	48.41	63.62	23.84
Bihar	8.41	13.01	20.57	25.97	37.4	46.24	56.89	21.94
Assam	1.52	3.63	12.14	19.76	32.08	45.86	56.62	16.18
Odisha	0.79	3.78	12.36	19.02	29.09	42.93	57.37	15.48
Uttar Pradesh	2.18	5.7	12.64	19.05	28.54	38.67	51.36	15.31
West Bengal	0.97	3.38	9.92	13.76	24.66	37.83	53.04	15.27

Note: All numbers are in percentages. Only those states where the percentage of women currently in menopause are 15 & above.

Among these states, Bihar stands out with the highest prevalence of premature menopause among women aged 30–39. Over 8.41% of women in this age group in Bihar are already experiencing premature menopause, compared to 5–5.5% in Andhra Pradesh and Telangana and a national average of just 2.36%. This disparity is even more pronounced among women aged 35–39, with over 13% experiencing menopause in Bihar compared to 11–12% in Andhra Pradesh and Telangana and a national average of 5.5%. The prevalence rates of premature menopause in Bihar are nearly four times the national average for the 30–34 age group and more than double the national average for the 35–39 age group.

### Sample

The background characteristics for each of the variables examined in the study across the different regions viz. India, Bihar, and Andhra Pradesh & Telangana is shown in Table 2. The study uses data from the NFHS-5 survey that comprises 191,087 women between the ages of 30 and 39.

**Table 2:** Respondent’s characteristics of women aged 30–39 years, NFHS-5 (2019–21).

VARIABLES	India	Bihar	Andhra Pradesh & Telangana
	Frequency (Percentage)		
Education Level			
No Education	54,600 (27.69)	5482 (53.98)	4567 (36.33)
Primary	28,890 (15.16)	1316 (13.40)	1419 (14.83)
Secondary	85,288 (43.83)	2605 (26.70)	4028 (38.39)
Higher	22,309 (13.32)	445 (5.92)	1120 (10.45)
Wealth Index			
Poor	79,896 (37.29)	6847 (66.99)	2759 (21.66)
Middle	39,593 (20.22)	1546 (15.69)	3532 (30.67)
Rich	71,598 (42.50)	1455 (17.32)	4843 (47.67)
Caste			
Scheduled Caste	36,123 (22.49)	2378 (23.32)	2410 (21.59)
Scheduled Tribe	35,144 (9.61)	347 (3.69)	862 (5.78)
Other Backward Classes	72,981 (45.07)	5532 (55.26)	6417 (55.66)
General	36,495 (22.06)	1526 (17.10)	1299 (16.57)
Don’t Know	1180 (0.77)	52 (0.64)	88 (0.40)
Religion			
Hindu	144,963 (81.94)	8662 (87.07)	9724 (84.47)
Muslim	22,439 (12.56)	1169 (12.77)	917 (8.73)
Others	23,685 (5.50)	17 (0.16)	493 (6.80)
Area of Living			
Urban	50,311 (34.59)	1159 (17.53)	3175 (34.19)
Rural	140,776 (65.41)	8689 (82.47)	7959 (65.81)
Age Group			
30–34	95,072 (49.81)	4950 (50.54)	5287 (47.76)
35–39	96,015 (50.19)	4898 (49.46)	5847 (52.24)
Children			
0	12,017 (5.53)	218 (2.56)	616 (5.69)
1–2	98,129 (53.88)	2208 (23.50)	7463 (69.25)
4–5	67,140 (33.67)	5178 (51.79)	2890 (23.80)
5+	13,801 (6.92)	2244 (22.15)	165 (1.27)
Age of women at First Birth			
< 18	33,083 (18.80)	2455 (25.27)	3316 (29.14)
18–21	58,108 (30.96)	3810 (37.41)	3482 (30.70)
22–24	45,763 (23.76)	2195 (22.54)	2144 (19.73)
25+	54,133 (26.48)	1388 (14.78)	2192 (20.44)
Age of women at Last Birth			
< 21 years	24,333 (14.48)	767 (8.09)	3352 (30.79)
22–24	40,561 (22.22)	1941 (19.55)	3012 (26.71)
25–28	59,539 (31.07)	3563 (35.96)	2604 (22.97)
29+	66,654 (32.33)	3577 (36.40)	2166 (19.53)
Contraceptive Usage			
No	50,956 (25.00)	2701 (29.54)	2496 (20.85)
Yes	140,131 (75.00)	7147 (70.46)	8638 (79.15)
Body Mass Index			
< = 18.5 kg/m2	20,927 (10.81)	1609 (15.70)	1358 (10.04)
18.5–24.9 kg/m2	109,522 (54.32)	5800 (57.70)	5557 (46.59)
> 25 kg/m2	60,638 (34.87)	2439 (26.59)	4219 (43.38)
Anemia			
No	89,605 (46.69)	3816 (40.40)	4863 (44.18)
Yes	101,482 (53.31)	6032 (59.60)	6271 (55.82)
Female Sterilization			
No	109,124 (54.25)	4769 (49.63)	2827 (22.92)
Yes	77,024 (45.75)	5043 (50.37)	8134 (77.08)
Health Insurance			
No	124,710 (67.56)	8528 (86.28)	3107 (25.98)
Yes	66,377 (32.44)	1320 (13.72)	8027 (74.02)

As evident from the table above, the distribution of women was similar across regions for socio-demographic variables, including social group, age, and religion. However, a higher proportion of women in India (13.32%) have completed higher education compared to those in Bihar (5.92%) and Andhra Pradesh & Telangana (10.45%). Additionally, a higher number of women from Bihar belong to the poorest wealth quintile (66.99%) compared to those from India (37.29%) and Andhra Pradesh & Telangana (21.66%). The number of women at the national level (42.50%) and in Andhra Pradesh & Telangana (47.67%) in the richest wealth quintile (17.32%) were approximately two-and-a-half times more than women in Bihar (Table 3).

Moving to reproductive history, a greater percentage of women in Bihar (62.68%) had their first child before the age of 21 compared to those at the national level (49.76%) and in Andhra Pradesh & Telangana (59.84%). Similarly, I observe a higher percentage of women having 5 or more children in Bihar (22.15%) as compared to national level (6.92%) and Andhra Pradesh & Telangana (1.27%). I also find differences in the history of contraceptive usage, with only 70.46% of the women in Bihar having ever used any method of contraception compared to 75% at the national level and 79.15% in Andhra Pradesh & Telangana.

Looking at the health indicators, I find that a higher percentage of women in Bihar (59.60%) have experienced anemia as compared to the national average (53.31%) and in Andhra Pradesh & Telangana (55.82%). The rate of sterilization in Bihar is almost 50% as compared to 46% at national level and 77% Andhra Pradesh & Telangana. Lastly, only 13% of women in Bihar have health insurance as compared to 32% at national level and 74% in Andhra Pradesh & Telangana.

### Hysterectomy & naturally induced menopause

**Fig. 1:**
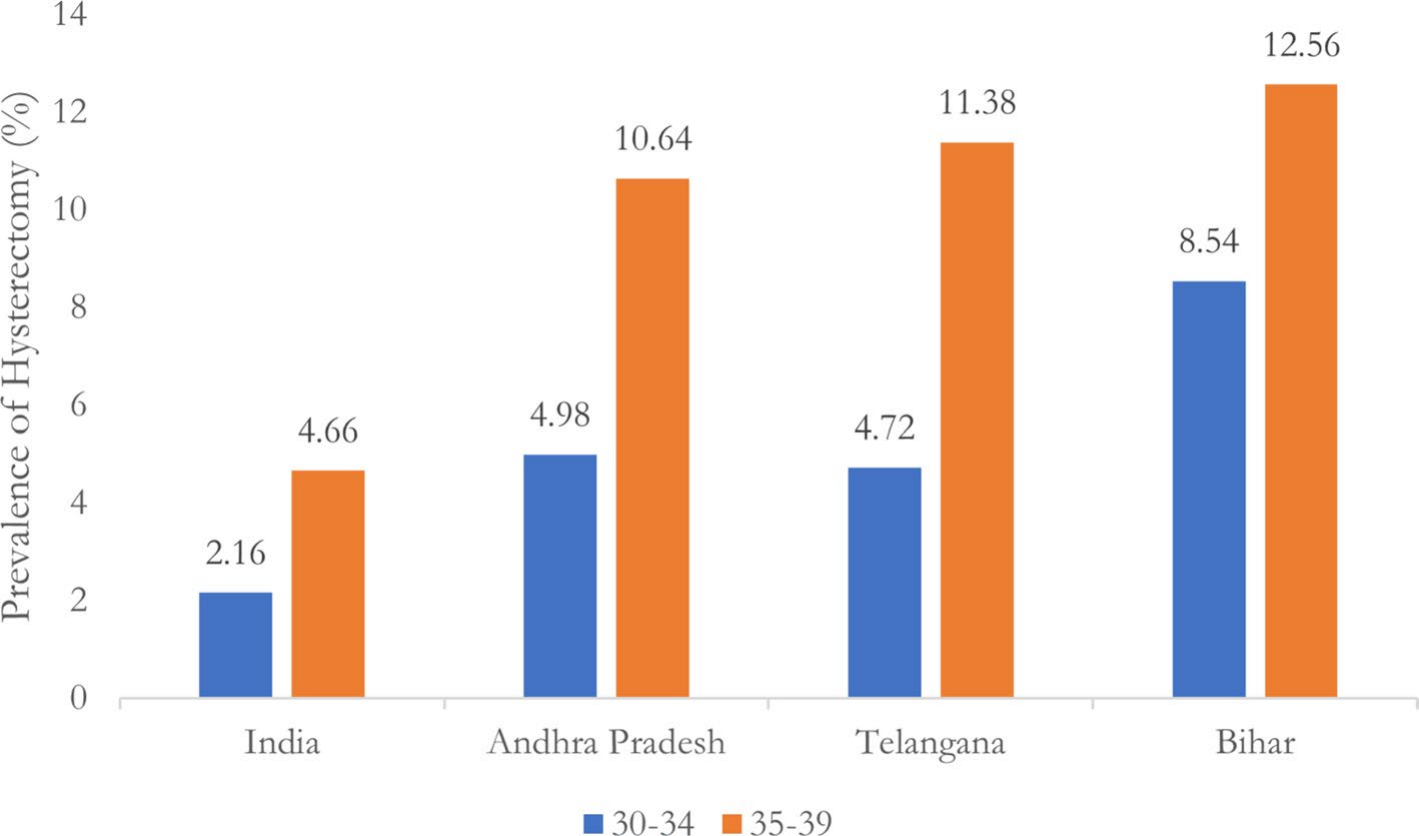
Prevalence of Hysterectomy among women aged 30-39 in India and the top 3 states, NFHS-5 (2019-21).

**Fig. 2:**
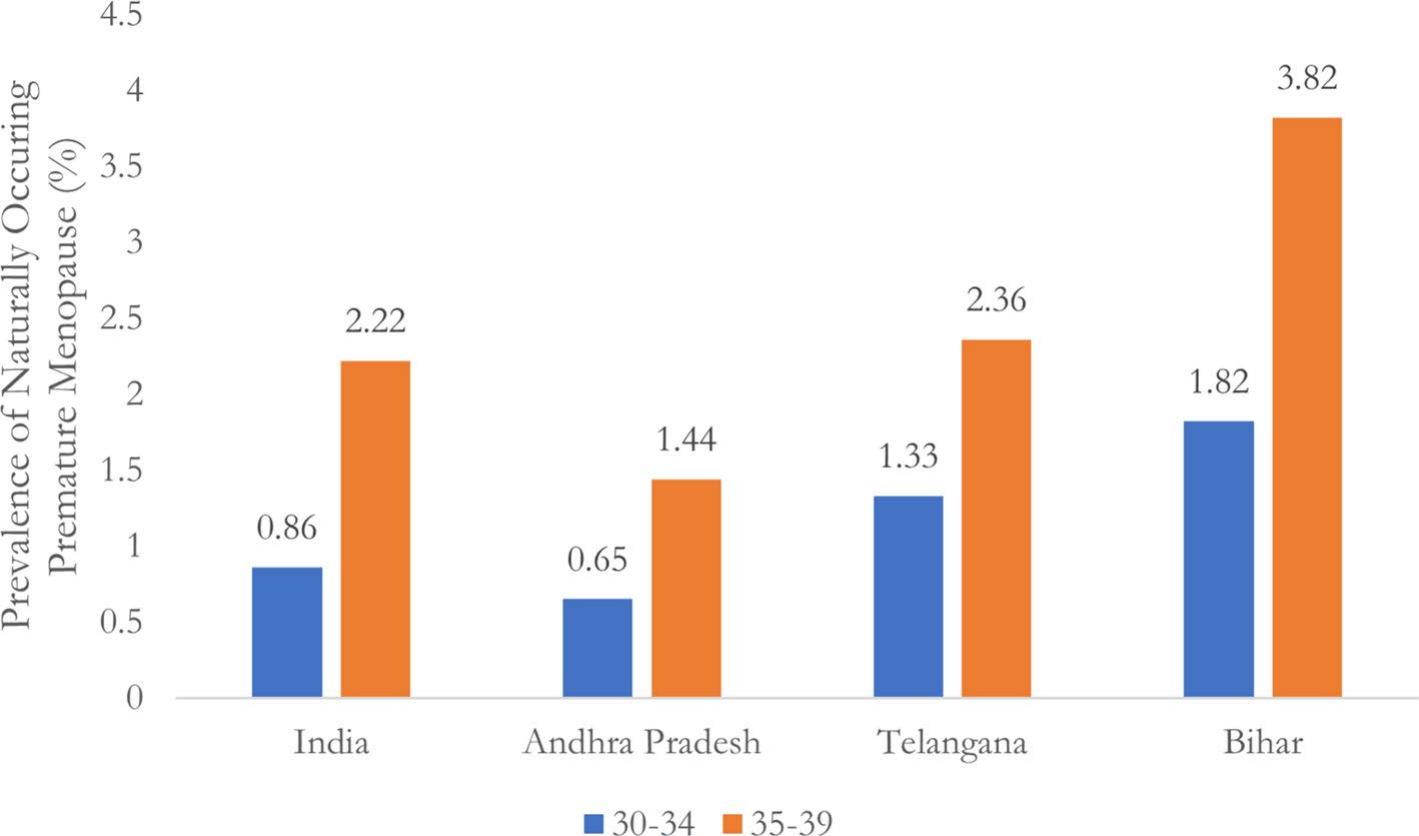
Prevalence of naturally occurring premature menopause among women aged 30-39 in India and the top 3 states, NFHS-5 (2019-21).

To disentangle the potential role of hysterectomy, I examined the prevalence of both hysterectomy-induced and naturally induced premature menopause. As shown in Fig. 1, hysterectomy rates in Bihar, Andhra Pradesh, and Telangana far exceed national averages, particularly among women in their 30 s. In Bihar, for instance, the hysterectomy rate among women aged 30–34 is a staggering 8.54%, nearly four times the national average. Figure 2 displays the prevalence of naturally induced premature menopause after excluding cases attributed to hysterectomy, demonstrating that even after accounting for hysterectomy, Bihar’s rate of premature menopause remains significantly higher than the national average, particularly in the 30–39 age group.

The disproportionately high rates of premature menopause in Andhra Pradesh and Telangana, while concerning, have been linked in previous studies to factors like the Aarogyasri health insurance scheme, which may incentivize hysterectomies^12,13^. However, the reasons behind Bihar’s alarming numbers, even after accounting for hysterectomies, remain largely unexplored. This demands an urgent investigation into the complex interplay of potential drivers.

### Results from binary logistic regression

To further explore the factors associated with premature menopause in Bihar, I conducted binary logistic regressions examining the predictors of both naturally induced and hysterectomy-induced premature menopause among women aged 30–39. Separate models were run for India as a whole, Bihar, and Andhra Pradesh/Telangana combined.

In Bihar, education emerged as a strong protective factor against naturally induced premature menopause. Women with at least a secondary education had significantly lower odds of experiencing menopause before age 40 compared to those with no education (OR: 0.505, *p* < 0.001). However, there were no significant effects for women with primary or higher education. Contraceptive use was also associated with lower odds of naturally induced premature menopause (OR: 0.059, *p* < 0.001). However, several reproductive factors increased the risk of naturally induced premature menopause in Bihar, including younger age at first birth, younger age at last birth, and having a greater number of children. Strikingly, female sterilization was significantly associated with a nearly five-fold increase in the odds of naturally induced premature menopause in Bihar (OR: 5.497, *p* < 0.001).

While lower education was consistently protective against naturally induced PM across all regions, the findings related to wealth differed. In India overall and in Andhra Pradesh/Telangana, women of higher socioeconomic status (as indicated by the wealth index) had lower odds of experiencing naturally induced premature menopause. However, wealth was not a significant predictor in Bihar. This suggests that other factors, potentially specific to Bihar, may be overriding the protective effect of socioeconomic advantage observed elsewhere.

Similarly, for hysterectomy-induced premature menopause, Higher education, later age at first birth, later age at last birth, and contraceptive use were all significantly associated with lower odds of hysterectomy-induced premature menopause in Bihar. Particularly noteworthy is that health insurance coverage (OR: 1.340, *p* < 0.001) was a significant risk factor for hysterectomy in Bihar. This suggests that access to insurance may be driving increased utilization of this procedure to deal with menstrual or uterine related problems, potentially due to financial incentives for providers or greater healthcare-seeking behavior among insured women. Female sterilization (OR: 13.68, *p* < 0.001) was also a significant risk factor, indicating a potential link between these two reproductive health outcomes.

A striking and consistent finding across both types of premature menopause in Bihar is the significant association with female sterilization. While sterilization is often promoted as a safe and effective method of family planning, the results suggest a potential link between this procedure and both naturally induced and hysterectomy-induced PM in this region. Further investigation is urgently needed to understand the underlying reasons for this association and explore potential long-term health implications (Table 3).

**Table 3:** Determinants for naturally induced menopause and hysterectomy among women aged 30–39 in India, Bihar, Andhra Pradesh & Telangana, NFHS-5 (2019–21).

	Naturally Induced Menopause			Hysterectomy		
	India	Bihar	Andhra Pradesh + Telangana	India	Bihar	Andhra Pradesh + Telangana
VARIABLES	(1)	(2)	(3)	(4)	(5)	(6)
Education Level						
No Education						
Primary	0.718***	0.791	0.882	0.837***	0.889	0.719***
Secondary	0.664***	0.505***	0.573***	0.740***	0.728**	0.622***
Higher	0.407***	0.491	0.426*	0.441***	0.208***	0.350***
Wealth Index						
Poor						
Middle	1.116*	0.747	0.933	1.183***	1.054	1.219**
Rich	0.953	0.809	0.887	1.203***	1.007	1.432***
Caste						
Scheduled Caste						
Scheduled Tribe	0.956	0.710	1.681**	0.906	0.839	1.460*
Other Backward Classes	1.039	0.744	1.128	1.165***	1.029	1.225*
General	1.035	0.850	1.022	1.136**	0.947	1.152
Don’t Know	0.839	1.354	0.633	1.174	1.118	1.634
Religion						
Hindu						
Muslim	0.842**	0.849	0.642	0.842**	0.764	0.593***
Others	0.876	##	1.488	0.853	1.621	0.853
Area of Living						
Urban						
Rural	1.072	1.162	1.158	1.387***	1.219	1.933***
Age Group						
30–34						
35–39	2.703***	2.336***	1.740***	2.139***	1.491***	2.297***
Children						
0						
1–2	0.445***	0.270**	0.193***	1.522***	11.61**	0.546*
4–5	0.494***	0.434*	0.219***	1.936***	16.98***	0.636
5+	0.563***	0.512	0.102***	1.978***	18.11***	0.570
Age of women at First Birth						
< 18						
18–21	0.721***	0.680**	0.509***	0.873***	0.860	0.702***
22–24	0.586***	0.646	0.517**	0.765***	0.746*	0.718**
25+	0.476***	0.380***	0.340**	0.477***	0.403***	0.581**
Age of women at Last Birth						
< 21 years						
22–24	0.755***	0.570**	1.071	0.779***	0.900	0.735***
25–28	0.641***	0.429**	0.840	0.574***	0.690**	0.435***
29+	0.422***	0.240***	0.493	0.298***	0.376***	0.217***
Contraceptive Usage						
No						
Yes	0.0958***	0.0585***	0.358**	0.0122***	0.0164***	0.0858***
Body Mass Index						
<= 18.5 kg/m^2^						
18.5–24.9 kg/m^2^	0.810***	1.035	0.885	1.065	0.995	1.129
> 25 kg/m2	0.867**	1.060	0.916	1.172***	1.230**	0.954
Anemia						
No						
Yes	0.809***	0.922	0.540***	0.641***	0.830**	0.441***
Female sterilization						
No						
Yes	3.195***	5.497***	1.595	25.19***	13.68***	7.069***
Health insurance						
No						
Yes	1.081	1.188	0.871	1.197***	1.340***	1.314***
Constant	0.0696***	0.839	0.174***	0.228***	0.0803**	0.227***
Observations	157,612	9,009	9,445	167,006	9,799	10,905

Col (1)–(3) shows the determinants of naturally induced menopause among women aged 30–39 in India, Bihar, and AP & Telangana, respectively. Col (4)–(6) shows the determinants for hysterectomy among women aged 30–39 in India, Bihar & Telangana, respectively.

***p.01, **p.05, and *p.1

## Discussion

This study has revealed a concerningly high prevalence of premature menopause among women in Bihar, India. These findings demonstrate that Bihar’s rates of premature menopause exceed national averages and those of other high-prevalence states, even after accounting for the influence of hysterectomies. Regression analysis identified several key factors associated with premature menopause in Bihar, including lower education, younger age at first birth, and, most strikingly, female sterilization.

The most striking and concerning finding in this study is the strong association between female sterilization and both naturally induced and hysterectomy-induced premature menopause in Bihar. It is important to clarify the distinction between these two types of premature menopause. *Naturally occurring premature menopause* refers to the cessation of menstruation before age 40 due to natural causes. *Hysterectomy-induced premature menopause* occurs when a hysterectomy, particularly one involving the removal of both ovaries (oophorectomy), leads to the premature cessation of menstruation and ovarian hormone production. Our findings indicate that female sterilization is associated with both of these outcomes in Bihar.

It’s important to note that female sterilization itself (*tubal ligation*) does not directly interfere with ovarian function or hormone production. Therefore, the high prevalence of premature menopause among sterilized women in Bihar is unlikely to be a *direct* result of the sterilization procedure itself. However, the quality and safety of sterilization procedures in the region could be an indirect contributing factor. Botched sterilizations may lead to complications such as pelvic inflammatory disease, chronic pain, or excessive bleeding, which, in turn, could necessitate hysterectomies. If these hysterectomies involve the removal of the ovaries (*oophorectomy*), they would directly induce menopause. It’s essential to acknowledge that, due to data limitations, we were unable to determine the specific type of hysterectomy (with or without oophorectomy) performed on women in our sample, which limits our ability to precisely quantify the contribution of hysterectomy to premature menopause. To mitigate this, our analysis also focused on naturally occurring premature menopause, which is independent of hysterectomy.

Several potential explanations for this association, beyond the direct impact of the procedure itself, warrant further investigation. Previous studies in India have suggested a link between sterilization and an increased risk of hysterectomy^14,15^. The high rates of female sterilization, often perceived as a straightforward means of family planning once a desired family size is reached, may inadvertently contribute to later hysterectomy needs due to complications like Pelvic Inflammatory Disease or other gynecological issues^14,16^. The fact that this study found sterilization to be a significant predictor of both naturally induced menopause and hysterectomy in Bihar further underscores the need to investigate this complex interplay.

A critical consideration is the potential for selection bias. Women who choose sterilization may differ systematically from women who choose other contraceptive methods or no contraception at all. They may have a higher prevalence of pre-existing gynecological conditions, face greater socioeconomic pressures, or have different reproductive health preferences. While I controlled for several socioeconomic factors in the analysis, unmeasured confounders could still contribute to the observed association. For instance, women experiencing early perimenopausal symptoms, such as irregular bleeding, might be more inclined to choose sterilization as a permanent method of contraception. This potential for reverse causality complicates our ability to conclude that sterilization *causes* premature menopause.

Beyond the direct impact of the procedure itself, the quality and safety of sterilization procedures in Bihar could be an indirect contributing factor to premature menopause. News reports have conducted exposés on Bihar’s sterilization camps where poor women have been sterilized in exchange for monetary compensation^17^. These exposés highlight the coercive tactics used by health officials to get women amenable to sterilization and the deplorable conditions under which the sterilizations were performed^17,18^. For impoverished women from rural areas, sterilizations are typically conducted in outpatient, government sterilization camps in temporary facilities such as small schools and public health clinics^19^. The conditions of these camps have long been criticized by scholars for their unsafe and unsanitary conditions^19,20^. The potential for coercion and lack of informed consent in these settings raises serious ethical concerns. This can be seen as a violation of women’s reproductive rights and bodily autonomy. The observed association between sterilization and premature menopause may reflect the long-term consequences of these unethical practices and the neglect of women’s reproductive health needs.

To address these ethical concerns, several policy reforms are needed. First, stricter regulations and monitoring of sterilization camps are essential to ensure adherence to safety standards and ethical guidelines. Second, healthcare providers need comprehensive training on informed consent, counseling, and the provision of a full range of contraceptive options, empowering women to make voluntary and informed decisions about their reproductive health. Third, increased investment in public health infrastructure and access to quality healthcare services is crucial to reduce the reliance on sterilization as the primary method of family planning and to address the underlying health issues that may contribute to the need for hysterectomies.

While the finding between sterilization and premature menopause is correlational and does not establish a causal link, it raises serious concerns about the potential for coercion and lack of informed consent in family planning programs. Scholars have argued that population control policies often disproportionately target women from lower socioeconomic backgrounds^21,22^. This targeting can result in the neglect of their individual reproductive needs and rights.

Beyond sterilization, these findings highlight the persistent influence of social determinants of health on women’s reproductive health in Bihar. Lower education was consistently associated with a higher risk of premature menopause, underscoring the importance of investing in girls’ education and women’s empowerment. Interestingly, wealth was not a significant predictor of premature menopause in Bihar, despite its effect in other regions of India. This suggests that other factors, potentially specific to Bihar, may be overriding the benefits of socioeconomic advantage. These factors might include caste discrimination in healthcare access, cultural norms around women’s health, limited access to quality healthcare in rural areas. In other words, socioeconomic advantage alone may not be sufficient to mitigate premature menopause risk when women face systemic gender inequalities and limited access to quality healthcare.

Future research should adopt an intersectional feminist lens to examine how the interplay of gender, caste, class, and other social identities shapes the risk of premature menopause and access to reproductive healthcare in Bihar. For instance, lower-caste women in rural areas may face greater barriers to accessing quality reproductive healthcare, increasing their risk of complications and, ultimately, hysterectomy. This approach can provide a more nuanced understanding of the complex factors contributing to the state’s high premature menopause rates.

The association between younger age at first birth and increased premature menopause risk is consistent with a growing body of research highlighting the long-term health consequences of early childbearing^23^. This finding underscores the need for adolescent reproductive health programs and expanded access to family planning services in Bihar.

The results also point to potential concerns regarding the utilization of hysterectomies. The finding that health insurance coverage is a risk factor for hysterectomy in Bihar suggests that access to insurance may be driving increased utilization of this procedure, potentially due to financial incentives for providers or greater healthcare-seeking behavior among insured women. Another possibility is that women with insurance are more likely to be diagnosed with gynecological problems, leading to a higher rate of hysterectomies. This warrants further investigation to ensure that hysterectomies are being performed only when medically necessary.

The negative association between anemia and both naturally induced menopause and hysterectomy was an unexpected finding. While the reasons for this are unclear, one possible explanation is that women with severe anemia may be considered less suitable candidates for elective surgeries like hysterectomy. Alternatively, severe anemia might be associated with lighter or less frequent periods, which could delay the self-recognition or reporting of menopause. Further research is needed to fully understand the complex relationship between anemia and menopause in this population.

The findings from the study all for a comprehensive and multifaceted public health response tailored to the specific needs of women in Bihar. This response must move beyond a narrow focus on hysterectomy and address the broader social, economic, and reproductive health factors driving premature menopause in this region. Priority actions include increasing investments in girls’ education and women’s socioeconomic empowerment, expanding access to adolescent reproductive health programs, and promoting a wider range of family planning options beyond sterilization. Specifically, this includes mandatory training programs for healthcare providers, public health campaigns, strengthening monitoring and regulation of sterilization camps and increased access to quality reproductive healthcare.

Moving forward, one needs to critically examine the quality and safety of sterilization practices in Bihar. This requires implementing stricter regulations and monitoring of sterilization camps, investing in training and resources for healthcare providers to ensure safe and ethical sterilization procedures, and empowering women with information about the potential risks and benefits of different family planning methods. Raising awareness among women and healthcare providers about the risks and long-term consequences of premature menopause, including the potential role of sterilization, is vital for enabling women to make informed decisions about their reproductive health.

While the findings from this study offer valuable insights into the drivers of premature menopause in Bihar, it is essential to acknowledge the limitations of the study. First, the cross-sectional nature of the NFHS-5 data prevents us from establishing causal relationships between the identified risk factors and premature menopause. Longitudinal studies are needed to better understand the temporal relationships and causal pathways involved. Second, as with all survey data, self-reported information on menopause status and other variables is subject to recall bias and social desirability bias, which could influence the findings. Third, the NFHS-5 dataset does not provide detailed information on the types of hysterectomies performed (*with or without oophorectomy*) or the specific methods of female sterilization used, or the types of contraceptives used (*hormonal, barrier, natural*). This limitation prevents us from fully disentangling the potential mechanisms linking these procedures to premature menopause. Additionally, we were unable to explore the role of certain factors that have been linked to premature menopause in other studies, such as smoking, genetic predisposition, and environmental exposures, due to data limitations. Future research incorporating these variables and using more detailed data on sterilization procedures, hysterectomy types and contraceptive methods would provide a more comprehensive understanding of the complex drivers of premature menopause in Bihar.

## Conclusion

In conclusion, this study highlights the concerningly high rates of premature menopause in Bihar and identifies a significant association with female sterilization. These findings underscore the need for a multifaceted public health response that addresses the social determinants of health, improves the quality and ethics of family planning services, and empowers women to make informed decisions about their reproductive health. Further research is needed to fully understand the complex interplay of factors contributing to premature menopause in Bihar and to develop effective interventions to improve women’s reproductive health and well-being.

## Data Availability

The dataset used in this study is publicly accessible and can be obtained upon request from the Demographic and Health Surveys (DHS) Program website at https://dhsprogram.com/data/dataset/India_Standard-DHS_2020.cfm?flag=0.
